# Multidisciplinary management in panfacial trauma: A clinical study

**DOI:** 10.6026/973206300221405

**Published:** 2026-03-31

**Authors:** Pallavi Karadiguddi, Prachi Dwivedi, Vijay Shekhar, Sree Ram Reddy, Khushboo Chhabaria Peswani, Ravinder Pal Singh Rana, Rahul Tiwari

**Affiliations:** 1 Department of Oral and Maxillofacial Surgery, KLE JGMM Medical College, Hubli, Karnataka, India; 2 Department of Oral and Maxillofacial Surgery, PGMO, District Hospital Shivpuri, Madhya Pradesh, India; 3 Department of Dentistry, Nalanda Medical College Hospital, Agamkuan, Patna, Bihar, India; 4 Department of oral and maxillofacial surgery, Sree Mithra Dental and MaxFace Specialists, Tanuku, Andhra Pradesh, India; 5 Department of Oral and Maxillofacial Surgery, RKDF Dental College and Research Centre, Bhopal, Madhya Pradesh, India; 6 Department of Dental Surgery, Command Military Dental Centre, Jaipur, Rajasthan, India; 7 Department of Oral and Maxillofacial Surgery, Narsinhbhai Patel Dental College and Hospital, Sankalchand Patel University, Visnagar, Gujarat, India

**Keywords:** Panfacial fracture, multidisciplinary team (MDT), airway management, virtual surgical planning (VSP), outcomes

## Abstract

Management of panfacial fractures lacks standardized multidisciplinary pathways and variability in coordination, surgical sequencing and airway planning may contribute to delays and postoperative complications. Therefore, it is of interest to assess if a structured multidisciplinary team (MDT) referral system leads to an improvement in process and outcome measures versus usual care where patients are referred sequentially. A comparison of a single-center cohort with two different treatment strategies; primary outcomes were time-to-definitive fixation and major postoperative complications. Secondary outcomes were airway strategy, prolonged hospitalization, reoperation rate and functional occlusal alignment at 3 months. MDT pathway was found to lead to reduction in time to definitive management and less complications advocating the use of structured-multidisciplinary workflow in panfacial trauma services.

## Background:

Panfacial fractures are high-energy and injurious to numerous facial subunits; associated airway, ocular and intracranial issues are common place, rendering treatment inherently team-oriented. Modern clinical series have stressed that stabilization is only a consideration with regard to fixation technique but also in the case of concomitant procedures, timing and responsible specialty input for minimizing secondary deformity and complications [1]. The sequence of surgical procedure is still under a matter of debate and the recent literature confirms that "bottom-up/top-down"concepts can be in close agreement with each other, except for very complex patterns where custom procedural sequence may be proposed based on dominant fragments and reliable reference units [2, 3]. Variation in provider pathways and specialization-defined care has also been associated with variations in complications on systematic review, supporting the case for standardization of practice [4]. Simultaneously, point-of-care virtual surgical planning (VSP) and three-dimensional (3D) printing are emerging in complex craniofacial trauma to improve reduction accuracy and operative efficiency along with team-based planning [5]. Therefore, it is of interest to report and compare clinical process measures and early outcomes of a structured multidisciplinary pathway versus non-pathway care in patients with panfacial trauma.

## Materials and Methods:

A comparative clinical cohort research was done at a tertiary trauma referral center from January 2022 to December 2024. An MDT pathway was started in January 2023, so outcomes were compared between standard care (pre-pathway) and MDT pathway care (post-pathway). Institutional audit approval was obtained and data were taken from prospectively maintained trauma logs and electronic medical records. Adults aged ≥18 years with operatively treated panfacial fractures (mandible with midface and/or upper face involvement) were included. Exclusion criteria were isolated single-region fractures, definitive treatment at another hospital, death within 48 hours due to non-facial causes, or incomplete documentation. The MDT pathway included coordinated assessment by maxillofacial surgery, anaesthesiology, trauma surgery, ophthalmology (and neurosurgery when needed), standardized airway planning, CT-based operative mapping and planned theatre access. Primary outcomes were time from admission to definitive ORIF and a composite major complication within 12 weeks (deep infection/plate exposure, clinically significant malocclusion needing reintervention, vision-threatening complication, or unplanned return to theatre). Secondary outcomes included airway approach, ICU and hospital length of stay and reoperation rate. Continuous variables were analyzed using Student's t-test or Mann-Whitney U test, categorical variables using χ^2^or Fisher's exact test and significance was set at two-sided p < 0.05.

## Results:

Baseline features between cohorts were similar, demonstrating good clinical comparability of standard care and MDT pathway groups. The majority of patients were young men and road traffic injury was the leading cause. Frequency of CT-confirmed traumatic brain injury and complex midrace patterns were comparable and orbital involvement was common in both groups, in line with the usual complexity of panfacial trauma. No baseline variable was statistically significant, thus it is less likely that outcome differences were biased by unmeasured initial case-mix Table 1 (see PDF). MDT reduced the time to definitive ORIF by a substantial amount and also raised the number of children treated within 5 days. MDT care demonstrated also greater utilization of sub mental intubation and CT-VSP/3D-assisted planning. The length of stay was significantly shorter in the MDT group. While the composite overall major complication rate was less with MDT care (numerically), this did not achieve statistical significance, "probably due to the low number of events relative to sample size". Favoured trends were also seen for reduced number of, infections, malocclusion events and unplanned returns to theatre Table 2 (see PDF).

## Discussion:

Current research suggests that structuring panfacial trauma management as an MDT pathway improves key process metrics. That included earlier definitive fixation, increased advanced planning utilization. This is associated with clinically meaningful reductions in LOS and complication trends. Airway strategy is a central MDT decision point in panfacial trauma. Current research's increased use of submental intubation aligns with contemporary evidence supporting submental routes as a pragmatic alternative when nasotracheal intubation is contraindicated and operative access precludes standard oral tubes [6, 7]. Comparative outcome data indicate submental intubation can reduce infection and hospitalization compared with tracheostomy in selected populations. This is consistent with current research's observed reduction in LOS and a numerical decline in tracheostomy rates [6, 8]. Because airway choice intersects with fracture pattern, operative sequencing and planned fixation stages, embedding anesthesiology early into the workflow likely contributed to improved coordination and theatre readiness [7, 9]. A second MDT-linked mechanism is surgical planning standardization. We observed significantly higher adoption of VSP/3D-assisted planning in the MDT cohort. Evidence from acute complex maxillofacial trauma supports CT-driven VSP to improve preoperative understanding of fracture anatomy, plate adaptation strategy and operative efficiency-particularly in comminuted, multi-subunit injuries [10, 11]. Likewise, broader evaluations of 3D printing in acute maxillofacial trauma describe benefits in pre-bending plates, improving spatial orientation and facilitating team communication. This is relevant to multidisciplinary planning meetings and shared mental models [12]. Current data cannot confirm whether VSP directly reduced complications. However, improved planning may reduce reduction errors that lead to malocclusion or secondary deformity and these outcomes trended lower in the MDT group. MDT pathways are also "referral hygiene"interventions- promoting expediency, clarifying responsibility and standardizing early consult triggers. Literature on quality improvement initiatives for structured facial injury referral networks indicate that the use of standardised processes can enhance completeness of assessment and optimise patient care. This is in keeping with the current pathway design and parallels the trend for time to definitive fixation [9]. Furthermore, modern studies of fracture configurations further substantiate that panfacial injury frequently presents concomitantly with complex mandibular, midface and cranial base-related issues highlighting why concerted decision- making is necessary [13, 14]. Rapid epidemiologic transitions and external system pressures (e.g., resource limitations, changes in trauma patterns) further support the need to maintain resilient MDT systems that endorse protocol zed care delivery [15]. Limitations are that this was a single center study, the relatively small number of patients and before/after type comparisons prone to temporal confounding such as staffing and theatre access. The composite measure of complications may be in need of larger comparison groups to draw safe conclusions. However, the demonstrated gains in timeliness, adoption of planning and LOS support the practical utility of MDT pathways as a system-level intervention for panfacial trauma care.

## Conclusion:

One MDT structured pathway (panfacial trauma) was related to more prompt definitive fixation, further utilization of predictive models and less time spent in hospital. Major complications were numerically less in the MDT group but results of a larger study would be required to determine an effect size. In general, standardized multi-disciplinary protocols may enhance efficiency and promote a safer, better coordinated reconstruction in challenging panfacial trauma.

## Advancement to Knowledge:

This clinical cohort study provides contemporary evidence (2020-2025 context) that implementation of a structured multidisciplinary team (MDT) pathway in panfacial trauma is associated with significantly reduced time to definitive fixation, increased adoption of VSP/3D-assisted planning, shorter hospital stay and favorable trends in complication rates compared with non-pathway care, thereby supporting protocolized MDT workflows as a system-level quality improvement strategy.
